# Perspectives and factors associated with informal human milk sharing: a mixed-methods systematic review protocol

**DOI:** 10.12688/hrbopenres.13718.1

**Published:** 2023-04-20

**Authors:** Niamh Vickers, Anne Matthews, Gillian Paul

**Affiliations:** 1 School of Nursing, Psychotherapy and Community Health, Dublin City University, Dublin, Leinster, D09Y8VX, Ireland

**Keywords:** informal human milk sharing, peer milk sharing, donor breastmilk, online milk sharing, mixed methods systematic review

## Abstract

**Background:** The practice of informal human milk sharing is a relatively new phenomenon and poses significant questions in the domain of infant feeding. Informal human milk sharing is a means of donating human milk from another lactating individual who is not the child’s biological parent, in a casual manner, that is, without the involvement of health professionals or agencies. The advent of digital technology has facilitated the donation and receipt of human milk through digital online platforms and thus has amplified this modern practice. This research aims to comprehensively examine and synthesize evidence about the motivations, barriers, facilitators and perspectives of individuals who both donate (donors) and the primary care givers of the infants who receive (recipients) human milk informally to provide to infants.

**Methods:** A mixed-methods systematic review will be undertaken. This review will consider qualitative, quantitative and primary mixed-methods studies which report on the factors associated with informal milk sharing, and on donors and recipients’ perspectives of the practice. Primary mixed-method studies will be included if the individual qualitative and quantitative components can be extracted. Three databases will be searched for studies on informal human milk sharing published from inception of the database. Study quality will be evaluated using the standardized JBI critical appraisal tools, selected based on the methodology in each individual study. Data extraction will be conducted using the JBI mixed methods data extraction form followed by data transformation, synthesis and integration. This mixed-methods systematic review will follow a convergent integrated approach in accordance with JBI guidance.

**Discussion**: Informal human milk sharing is a novel practice in the domain of infant feeding. This review will enable a thorough understanding of this practice from both the donors and recipients’ perspective and will have implications for healthcare professionals, policy and future clinical decision-making.

**Protocol registration number:** PROSPERO CRD42023405653

## Introduction

Breastfeeding is one of the most beneficial public health endeavours to optimize child health and survival ( North *et al.*, 2022). The World Health Organization (WHO) advocates the early initiation of breastfeeding at birth, exclusive breastfeeding until 6 months and the continuation of breastfeeding along with complimentary foods until 2 years of age and beyond ( WHO, 2021). Breastfeeding is an integral component of many of the Sustainable Development Goals (SDGs) which are espoused by all member states of the United Nations (UN). The SDGs comprise of 17 interlinked global goals intended to create a healthier and more sustainable world. It has been affirmed that breastfeeding is crucial to achieving many of the SDGs which aspire to end poverty, protect the planet and ensure peace and prosperity for all ( Holden *et al.*, 2017; United Nations, 2022). Breastfeeding has numerous recognised benefits which span individual, societal, environmental and economical domains. Human milk itself is a complex, individual-specific bodily fluid which is dynamic. It is characterised by vast individual variability pertaining to nutritional composition and bioactive constituents ( Mosca & Giannì, 2017). Correspondingly, scientific research has confirmed that human milk is the perfect, unparalleled source of nutrition for infants. Therefore, the need to position breastfeeding as a global public health priority is acknowledged. Globally, only 44% of infants aged 0–6 months are exclusively breastfeed ( WHO, 2021), which is suboptimal given the multiplicity of benefits derived from breastfeeding. Notably, there is often a disparity between the intention to breastfeed and the actuality as often the barriers to breastfeeding transcend beyond individuals and encompass social, cultural and political tiers ( Brown, 2017).

It is widely acknowledged that there is no one universally accepted definition or explicit meaning of the term ‘breastfeeding’. Breastfeeding is a complex process and comprises a multitude of meanings and dimensions. Notably, it has been articulated that there is a lack of accuracy and consistency in uniformly defining breastfeeding ( Labbok & Krasovec, 1990). Consequently, the term ‘breastfeeding’ alone falls short of adequately describing and defining the many infant feeding behaviours which incorporate the provision of breastmilk to an infant. However, the term ‘breastfeeding’ is typically recognised as being direct feeding of an infant at the breast ( Rosenbaum, 2022). Nevertheless, this term can also pertain to a variety of other practices which can include: combination feeding which is also recognised as non-exclusive feeding where an infant receives both breastmilk and a commercial milk formula (CMF), and exclusive breast milk pumping whereby the infant receives only breastmilk expressed from their mother. More than three and a half decades have elapsed since it was acknowledged that the term ‘breastfeeding’ alone was inadequate to precisely depict the multitude of breastfeeding behaviours that are recognized within the domain of ‘breastfeeding’ ( Labbok & Krasovec, 1990). At this juncture, The Interagency Group for Action on Breastfeeding devised specific categories where standardised and consistent terminology relating to outlining and categorising breastfeeding ( Labbok & Krasovec, 1990).

It has been stated that when breastfeeding is unsuccessful or cannot be undertaken, parents may choose to exclusively pump or provide CMFs ( Rosenbaum, 2022). Some infants in paediatric or neonatal intensive care units (PICU/ NICU) may receive donated human milk which is medically prescribed and provided by a milk bank when a mother’s own milk (MOM) is not available. Human milk banks (HMB) play a fundamental role in the provision of donor human milk (DHM) to infants who cannot otherwise receive their mother’s own human milk (MOM) ( Haiden & Ziegler, 2016). DHM is predominately reserved for infants born preterm where it has been widely affirmed that DHM as a substitute for MOM has instrumental benefits including improving clinical outcomes and reduction in the two central causes of mortality in this vulnerable cohort, namely sepsis and necrotising enterocolitis ( Cortez *et al*., 2018; Miller *et al*., 2018; Patel & Kim, 2018; Tran *et al.,* 2020). HM is beneficial for the health of infants as clinical practices have demonstrated that numerous beneficial components are preserved following pasteurization of the human milk ( Peila *et al.*, 2016). What is more, the provision of human milk to infants reduces the risks associated with CMF feeding such as otitis media, asthma, types 1 and 2 diabetes, atopic dermatitis, and infant hospitalization secondary to lower respiratory tract diseases ( McNiel *et al.*, 2010). There is global agreement that MOM is the preferred source of nutrition for infants and if not available pasteurized donor human milk (DHM) from an established milk bank is the preferable choice ( Daniels *et al.*, 2017; ESPGHAN Committee on Nutrition *et al.*, 2013; Pound *et al.*, 2020; WHO, 2011). In recent years, an alternative means of providing human milk to an infant is when parents undertake the practice of informal human milk sharing (IHMS) which comprises receiving donor human milk via informal means without the use of a milk bank ( Rosenbaum, 2022).

The practice of human milk sharing is not a new entity and has origins in antiquity. Accordingly, this practice has evolved from an alternative feeding practice for ‘need’, whereby this practice was essential for infant survival to contemporary times where substitutes are available, such as CMFs and donated non-maternal human milk. The sharing of milk derived from the practice of ‘wet nursing’ whereby infants were breastfed by a lactating woman who was not the biological mother of the infant. This ancient practice and alternative means of feeding has evolved considerably from being a necessity (2000BC) for infant survival, to a substitute of choice (950BC to 1800AD) ( Stevens *et al.*, 2009). This phenomenon of human milk sharing has progressed further and diffused from the practice of wet nursing to the sharing and receiving of pumped breastmilk via milk banking, and further again to involve the practice of informal, ‘peer-to-peer’ human milk sharing. The interchangeable terms for human milk sharing are noted as ‘peer to peer milk sharing’ and ‘informal human milk sharing’ as this practice is dependent on the donors and recipients to collaborate and agree the specific terms of the exchange ( Palmquist *et al.*, 2019). Human milk sharing is described in the literature as the voluntary, altruistic and commerce-free trading of human milk for infant feeding, outside of the milk banking system ( Peregoy *et al.*, 2022). In recent years, ‘for-profit’ human milk banks have been established . These private entities operate a business where breastmilk is purchased from a lactating woman and then processed and sold to hospitals ( UNICEF, 2021). Additionally, various online platforms and internet sites facilitate the commercial sale of human milk by individuals who are expressing milk and can advertise specific details such as images and the cost of human milk per volume ( Steele *et al.*, 2015).

The advent of digital technology and advancement of social media has had an explicit influence on informal milk sharing among people in online communities, where more information relating to this practice has become available in recent years ( Akma Jamil *et al.*, 2021). Additionally, parallel advancements in relation to refrigeration and high-quality, double electric breast pumps have also facilitated the increased impetus in this modern practice ( Peregoy *et al.*, 2022). Consequently, over the past decade, research into the practice of online breastmilk sharing has become more prevalent corresponding with the advancement in digital and online networks, internet enabled smart phones and social networking apps ( Akma Jamil *et al*., 2021; Noyes, 2020).

In recent years, there has been an increasing number of studies exploring various areas relating to the practice of informal human milk sharing (IHMS), but at present, there is no existing or ongoing mixed-method or individual systematic review on the subject. Preliminary searches of CINAHL (EBSCOhost), Scopus (Elsevier), and MEDLINE have been undertaken and no systematic reviews on the topic were identified. There have been two recently published scoping reviews which have mapped the existing literature on the practice of informal human milk sharing in the United States of America and globally, respectively ( Akma Jamil *et al*., 2021; Kullmann *et al.*, 2022). These prominent reviews in the field of IHMS have provided the impetus to enrich the breadth and depth of understanding of this contemporary practice. Thus, there is a justification for undertaking a mixed methods systematic review (MMSR) on this subject to efficiently integrate the existing evidence with the intention of providing explicit information including whether findings are consistent within the literature and can be generalized across settings and populations, as well as informing healthcare decision making in the future ( Büchter *et al.*, 2020) ( Mulrow, 1994). This MMSR will provide a comprehensive and methodological synthesis of the broader topic of IHMS and more specifically include the perspectives of donors and recipients and the factors associated with the practice. This review will also guide future research on the topic of IHMS.

## Study aim and review questions

A mixed-methods systematic review (MMSR) is proposed to evaluate the quantitative, qualitative and primary mixed-method studies pertaining to the practice of informal human milk sharing. The following questions aligned to the review question are:

•What are the factors (motivations, barriers and facilitators) associated with the practice of informal human milk sharing?

•What are the perspectives of recipients and donors on informal human milk sharing?

The findings of this review will be of value to researchers, healthcare professionals and policy makers. It will develop a richer understanding of the factors which are associated with this practice and it will enrich our understanding of the subjective perspectives of both donors and recipients of informal human milk sharing.

## Methods

### Inclusion criteria

This MMSR protocol has incorporated a PICo framework (Population, phenomenon of Interest, COntext) as recommended by JBI for (MMSR) ( Stern *et al.*, 2020), and this is visually represented below. The PICo framework ( Table 1) facilitated the identification of the defining characteristics for the inclusion criteria for the MMSR.

**Table 1.** PICo framework.

**Population:** donors and recipients of informal human milk sharing
**Interest:** motivations, barriers, facilitators, perspectives of informal milk sharing
**Context:** globally

**Population:** The review will consider studies that include donors and/or recipients of informal human milk sharing. While the recipient of the human milk will be an infant, for the purposes of this MMSR the term recipient refers to the individual who sourced the human milk for the infant and in most studies this will be a mother, father, primary care giver, or biological/ non biological parent. Studies which include donors and/or receivers of human milk via milk banks will not be included. Additionally, studies which incorporate other means of milk sharing, specifically: formal and or/informal milk sharing with financial compensation will not be included as well as studies which include other means of milk sharing such as wet nursing. Studies that look at clinical outcomes of infants who are provided with human milk either from milk banks or by an informal means will not be included. Additionally, research which documents an exploration of perspectives from individuals other than recipients or donors of human milk will not be included.

**Phenomena of interest:** This review will consider studies which report on the concept of informal human milk sharing, specifically on donors and recipients human milk via an informal means and the factors associated with this practice, including the motivations, facilitators and barriers to this practice.

**Context:** This review will consider studies from any geographic location globally.

**Type of studies:** This MMSR will consider qualitative, quantitative, and primary mixed-methods study designs for inclusion. Primary mixed-methods studies will only be taken into consideration if it is possible to explicitly extract the data from the quantitative or qualitative components ( Lizarondo *et al.*, 2022). The justification for this is founded on the JBI methodological guidance for MMSR which proposes that the disaggregation of quantitative data will need to be completed in primary-mixed methods studies ( Stern *et al.*, 2020). In this instance, the disaggregated quantitative data will then be qualitized in order for the integration to occur. If primary-mixed methods studies are presented already in an integrated format and disaggregation cannot be conducted, these studies will be excluded. Included studies will comprise of peer-reviewed, published, full text articles and grey literature sources. This MMSR will consider all languages and year of publication. The reference lists of articles attained will be manually searched for additional, pertinent papers. 

The justification for conducting a MMSR is to develop contextual understanding on the topic and to enhance credibility through the amalgamation of diverse categories of evidence ( Hong *et al.*, 2018). Additionally, conducting a MMSR will enable the enhancement, depth and richness of comprehension to facilitate an inclusive understanding of the evidence ( Hong *et al.*, 2018). It will enable a comprehensive insight into the practice as well as ascertaining if the findings and evidence from the literature confirms, supplement or contradict one another, thus facilitating a rich and broad perspective on the subject of informal human milk sharing from a global perspective.

This proposed MMSR will be conducted in accordance with the JBI methodology guidance for conducting MMSRs as outlined in Chapter 8: Mixed methods systematic reviews in the JBI Manual for Evidence Synthesis ( Aromataris & Munn, 2020). The protocol has been submitted and accepted for registration on International Prospective Register of Systematic Review PROSPERO CRD42023405653. This MMSR will employ a convergent integrated approach as articulated in JBI guidance which includes data transformation and enables reviewers to merge quantitative and qualitative data ( Stern *et al.*, 2020). This is the preferable approach, as the review question can be addressed by both research paradigms. This MMSR will follow the recommendations from JBI for reviewers following a convergent integrated approach which is the qualitisation of quantitative data, where quantitative data will be transformed and converted into textual descriptions ( Aromataris & Munn, 2020). This will enable the converted data to be compiled together and thus facilitate integration ( Stern *et al*., 2020).The mixing of data by the means of integration is a distinguishing feature of a MMSR and the appropriate integration is an instrumental defining characteristic of this type of systematic review ( Stern *et al.*, 2020).

### Search strategy

A comprehensive search strategy will aim to discover both published and unpublished studies. A three-phase process will be utilized for the search strategy as recommended by JBI. First, a preliminary search of CINAHL (EBSCOhost), Scopus (Elsevier), and MEDLINE was undertaken incorporating key terms. These databases were selected due to their dependability and availability across an extensive range of subject areas. Following this, an analysis of key words/terms in the titles, abstract and index terms used to describe the articles obtained in initial preliminary search will enable a more comprehensive search strategy to be developed. The following databases will be searched from inception to date: CINAHL (EBSCOhost), Scopus (Elsevier), MEDLINE and Embase (OvidSP). Subsequently, the reference lists for all studies selected will be manually examined for additional studies which may be included. Boolean operators and controlled vocabulary terms will be used as required for each database search. An additional search of grey literature will be completed based on the key terms used for the database search. This will include searching Google Scholar, Web of Science, Open Grey, Dissertation Abstracts International. It has been acknowledged that the inclusion of grey literature is vital to ensure a fully comprehensive systematic review.

### Study selection

Following the search, all identified citations will be loaded into Zotero Citation Management System version 6/2022. These will be imported to Covidence. JBI recommends the incorporation of Covidence (reviewed in the October 2018 issue of the Journal of the Medical Library Association) for the screening process and then uploading the final studies into SUMARI for the continuation of the review process. Titles and abstracts will then be reviewed in Covidence by two independent reviewers for assessment against the inclusion criteria for the review. The full text of selected citations will be assessed in detail against the inclusion criteria by two independent reviewers. Each full text study which is excluded will be documented and reported in the systematic review. Any discrepancies that develop between the reviewers will be settled through discussions or with the help of a third independent reviewer where this cannot be resolved ( Aromataris & Munn, 2020). The results of the search will be reported in full in the final review and presented in a Preferred Reporting Items for Systematic reviews and Meta-Analyses (PRIMSA) flow diagram ( Page *et al.*, 2021). The contemporary 27-item PRISMA checklist updated in 2020 and published in 2021 will be used ( Page *et al.*, 2021).

### Assessment of methodological quality

The final studies included will be appraised and evaluated for methodological quality. Quantitative and qualitative and the relevant components of mixed-methods studies which are selected for retrieval will be assessed by two independent reviewers for methodological quality using standardised critical appraisal instruments from JBI SUMARI, dependent on study design ( Aromataris & Munn, 2020). Using the appropriate standardized critical appraisal tools from JBI SUMARI, the methodological quality of mixed-methods studies will be evaluated in connection to the qualitative components and quantitative components. If required, relevant authors of papers will be contacted to seek clarification or to request any missing data ( Aromataris & Munn, 2020). Any disagreements that may transpire between the reviewers will be resolved through discussion or in consultation with a third reviewer. Following the critical appraisal, the results will be documented in descriptive format and visually presented in table format. Data extraction and synthesis will be performed on all studies, regardless of the results of their methodological quality results, where feasible ( Aromataris & Munn, 2020). Grey literature will be appraised and evaluated by using the (Authority, Accuracy, Coverage, Objectivity, Date, Significance) AACODS checklist which was developed specifically for use with grey literature resources ( Tyndall, 2010).

### Data extraction

Quantitative and qualitative data will be extracted from all studies included in the review by two independent reviewers using the standardised JBI data extraction form for MMSR following a convergent integrated approach ( Aromataris & Munn, 2020). Data extraction forms are the fundamental connection between the primary research studies and the systematic review and therefore a preliminary pilot test by two reviewers will be undertaken prior to the full systematic review ( Büchter *et al.*, 2020). Specific information about the populations, research techniques, phenomena of interest, context, and results relevant to the review questions will all be included in the data that is extracted. The following information will also be included in the data extraction process: study design, methods, sampling technique, inclusion and exclusion criteria, study setting, geographic location, and significant findings aligned to the research questions. The quantitative data will be the results of statistical tests undertaken, which will be either descriptive and/or inferential, depending on data ( Aromataris & Munn, 2020). The degree of credibility of qualitative data will be determined by how closely the findings match the themes and/or subthemes and the corresponding illustrations ( Aromataris & Munn, 2020). JBI guidance recommends three levels of credibility: unequivocal, credible and not supported ( Aromataris & Munn, 2020). The foundation for evaluating, exploring, summarising, and elucidating the body of evidence is foremost initiated by data extraction forms, which are the vital link between primary original research and systematic reviews ( Büchter *et al.*, 2020). As a result, their creation, trial testing, and use are fundamental steps in the process of conducting systematic reviews.

### Data transformation

Following extraction, quantitative data will then be transformed into ‘qualitized’ data. This process will involve the transformation of quantitative data into textual descriptors or narrative interpretation of the quantitative results, so as to respond directly to the review questions. This process is recommended by JBI as it is less error-prone than assigning numerical values to qualitative data, termed quantitization ( Stern *et al.*, 2020).

### Data synthesis and integration

This review will follow a convergent integrated approach in accordance with the JBI methodology for MMSRs ( Aromataris & Munn, 2020). This will comprise the assembling of the transformed, qualitized data with the qualitative data. Following this, the assembled data are then categorised and combined and integrated together based on similarities in meaning to create a set of integrated findings in the form of line of action statements.

### Study status

This MMSR protocol has been developed and registered on PROSPERO (CRD42023405653). We are currently completing the systematic search which will be followed by screening, data extraction, data synthesis and integration as outlined above.

## Discussion

Informal human milk sharing is a novel practice which is gaining momentum in recent times. This MMSR will facilitate a comprehensive understanding of this practice from both the donors and receivers of human milk carried out in an informal manner. This MMSR will help us gain a deeper understanding of the factors that contribute to this practice as well as the perspectives of both donors and receivers of informal human milk sharing. It has been documented that MMSRs can augment the capability of findings and contribute to policy and clinical decision making ( Stern *et al*., 2020). It is anticipated that the findings from this MMSR will be beneficial to healthcare professionals, researchers and policy makers, as well as providing a robust and comprehensive understanding of this practice from various perspectives.

## Dissemination

This MMSR will be submitted for publication in scientific journals and will be made publicly available as open access.

## Data Availability

No data are associated with this article. Zenodo: Preferred Reporting Items for Systematic Review and Meta-Analysis Protocols (PRISMA-P) checklist for “Perspectives and factors associated with informal human milk sharing: a mixed-methods systematic review protocol”. https://doi.org/10.5281/zenodo.7790311 ( Vickers *et al.*, 2023). Data are available under the terms of the Creative Commons Attribution 4.0 International License (CC-By 4.0).
