# Supplementary material for: Perspectives and factors associated with informal human milk sharing: a mixed-methods systematic review protocol
Source: HRB Open Res. 2023 Oct 25;6:24. Originally published 2023 Apr 20. [Version 2] doi: 10.12688/hrbopenres.13718.2 (PMC10472069; doi:10.12688/hrbopenres.13718.2)
Supplement: Peer review history

# Reviewer report: Reviewer response for version 2

**DOI:** 10.21956/hrbopenres.15132.r36763

**Published:** 2023-11-02

**Authors:** Kaitlin Drouin

**Affiliations:** 1 Brigham & Women’s Hospital, Boston, USA

No new comments. Looking forward to reading about your results in a future publication.

Is the study design appropriate for the research question?

Yes

Is the rationale for, and objectives of, the study clearly described?

Yes

Are sufficient details of the methods provided to allow replication by others?

Yes

Are the datasets clearly presented in a useable and accessible format?

Not applicable

Reviewer Expertise:

Infant feeding practices, infant nutrition and growth, human milk, donor human milk.

I confirm that I have read this submission and believe that I have an appropriate level of expertise to confirm that it is of an acceptable scientific standard.

# Reviewer report: Reviewer response for version 2

**DOI:** 10.21956/hrbopenres.15132.r36764

**Published:** 2023-10-25

**Authors:** Elizabeth J. O'Sullivan

**Affiliations:** 1 School of Biological and Health Sciences, Technological University Dublin, Dublin, Leinster, Ireland

Thank you to the authors for their review and incorporation of feedback. I look forward to the ultimate publication of this mixed-methods systematic review.

Is the study design appropriate for the research question?

Yes

Is the rationale for, and objectives of, the study clearly described?

Yes

Are sufficient details of the methods provided to allow replication by others?

Yes

Are the datasets clearly presented in a useable and accessible format?

Not applicable

Reviewer Expertise:

Nutrition, breastfeeding, infant feeding, breast milk expression, breast milk sharing.

I confirm that I have read this submission and believe that I have an appropriate level of expertise to confirm that it is of an acceptable scientific standard.

# Reviewer report: Reviewer response for version 1

**DOI:** 10.21956/hrbopenres.15005.r35186

**Published:** 2023-08-31

**Authors:** Misra Abdulahi

**Affiliations:** 1 Jimma University, Jimma, Oromia, Ethiopia

I am glad to review this well-written and timely manuscript. With this review, the authors aim to provide evidence on the perspectives of donors and recipients and the factors associated with the practice to guide healthcare professionals, policy and future clinical decision-making. The rationale of the review is clearly described and they have used a mixed methods systematic review which is a method that provides a complete basis for decision making compared to a single method review. The authors have clearly indicated that they will follow a convergent integrated approach in accordance with JBI guidance which includes data transformation and the merging of quantitative and qualitative data.

 I have a few editorial comments:

**Abstract**

- Background – care gives should be written in one word as caregivers.
- Methods – rewrite “…and on donors…” as “…and on donors’…”
- Discussion – rewrite “…both the donors...” as “…both the donors’...”

**Introduction**

- Informal human milk sharing is used several times in the texts. Once it is abbreviated in the introduction section, better to use the abbreviated one after that.
- Page3, paragraph 2 – The sentence “Nevertheless, this term can also pertain to a variety of other practices which can include: combination feeding which is also recognised as non-exclusive feeding where an infant receives both breastmilk and a commercial milk formula (CMF), and exclusive breast milk pumping whereby the infant receives only breastmilk expressed from their mother.” needs citation.
- Page 3 paragraph 3 – does “MOM” stand for mother’s own milk or mother’s own human milk? Be consistent.
- Page 3 paragraph 3 – does HM stand for human milk? If so, it has to be written in full when it first appears in the text.
- WHO appears only once. Thus it is better to avoid the abbreviation.
- Page 3 paragraph 4 - The sentence “Accordingly, this practice has evolved from an alternative feeding practice for ‘need’, whereby this practice was essential for infant survival to contemporary times where substitutes are available, such as CMFs and donated non-maternal human milk.” needs citation.
- Page 3 paragraph 4 - The sentence “The sharing of milk derived from the practice of ‘wet nursing’ whereby infants were breastfed by a lactating woman who was not the biological mother of the infant.” needs citation.
- Page 3 paragraph 4 - The sentence “This phenomenon of human milk sharing has progressed further and diffused from the practice of wet nursing to the sharing and receiving of pumped breastmilk via milk banking, and further again to involve the practice of informal, ‘peer-to-peer’ human milk sharing.” needs citation.
- Page 3 paragraph 4 - The sentence “In recent years, ‘for-profit’ human milk banks have been established.” needs citation.
- Page 3 paragraph 1, line 10  – “The SDGs comprise of  17 …” remove “of”
- Page 3 paragraph 1, line 23 – change “acknowl-  edged”  to “acknowledged”
- Page 3 paragraph 2, line 2 – change “breast- feeding” to “breastfeeding”
- Page 3 paragraph 2, line 13 - change “combina-  tion” to “combina tion”
- Page 3 paragraph 3, line 3 – change “paedi- atric” to “paediatric”
- Page 3 paragraph 3, line 19 – change “…to collaborate and agree…” to “…to collaborate and agree on…”
- Page 4 paragraph 2, line 1 –  change “…advancement…” to “…the advancement…”
- Page 4 paragraph 2, line 11 – “smart phones” has to be one word “smartphone”

**Methods**

- Page 4 paragraph 7, line 6 – “care giver” should be one word “caregiver”
- Page 4 paragraph 8, line 2 -  “shar-  ing” should be written as “sharing”
- Page 4 paragraph 8, line 3 -  “human” should be “of human”
- Page 5 paragraph 2, line 1 -  correct “…contextual understanding on…” as “…a contextual understanding of…”
- Page 5 paragraph 2, line 8 - change “ascertaining” to “ascertain”
- Page 5 paragraph 2, line 9 – change “confirms” to “confirm”
- Page 5 paragraph 5, line 1 – change “key words” to “keywords”
- Page 5 paragraph 5, line 1 – rewrite “…Dissertation Abstracts International…” “…and Dissertation Abstracts International…”
- Page 5 paragraph 6, line 17 – rewrite PRIMSA as PRISMA
- Page 5 paragraph 7, line 1 – rewrite “…and in consultation…” as “…and consultation…”
- Page 6 paragraph 1, line 3 – rewrite “…using the (Authority, Accuracy, Coverage, Objectivity, Date, Significance) AACODS checklist…” as “…using the AACODS (Authority, Accuracy, Coverage, Objectivity, Date, and Significance) checklist…”
- Page 6 paragraph 1, line 15 – rewrite “…aligned to the research questions.” As “…aligned with the research questions.”
- Page 6 paragraph 1, line 12 – rewrite “…as well as providing…” as “…as well as provide…”

Is the study design appropriate for the research question?

Yes

Is the rationale for, and objectives of, the study clearly described?

Yes

Are sufficient details of the methods provided to allow replication by others?

Yes

Are the datasets clearly presented in a useable and accessible format?

Not applicable

Reviewer Expertise:

maternal health, child health, breastfeeding, breast milk

I confirm that I have read this submission and believe that I have an appropriate level of expertise to confirm that it is of an acceptable scientific standard.

# Reviewer report: Reviewer response for version 1

**DOI:** 10.21956/hrbopenres.15005.r35183

**Published:** 2023-08-31

**Authors:** Mariana González de Oliveira

**Affiliations:** 1 Neonatal Medicine, University of Health Sciences of Porto Alegre - UFCSPA, Porto Alegre, Rio Grande do Sul, Brazil

I greatly appreciate the chance to engage with this thoughtfully written paper, presenting a well-timed and captivating overview. The paper outlines a meticulous protocol for an upcoming mixed-methods systematic review, aimed at comprehensively capturing and consolidating insights into the diverse populations, interests, and contextual aspects surrounding the informal sharing of human milk within existing scholarly works. Undoubtedly, this research subject holds significant promise in enriching our comprehension of this dynamic and enduring phenomenon.

 I have some minor observations that may be useful for the authors.

**Introduction:**

 Although breastfeeding terminology may not be consistently used in literature, there are some references to refer to, such as Boss’s et al. ^1^ and CDC’s glossary.

 In the second sentence, please change “complimentary” to “complementary”.

 I suggest looking at Cesar Victora’s papers which have published pioneer and extensive work on breastfeeding benefits for infants.

 I suggest changing the last sentence to “may”, instead of “will”: it may give an idea that the authors already know the results of a review that is about to be done. The same may apply to the statements under “study aim and review questions”.

**Methods:**

 Although the authors have chosen to exclude wet nursing, maybe you should consider including this practice in your revision.

 As made very clear, this proposed MMSR will be conducted in accordance with the JBI methodology, please consider justifying why.

 In the search strategy, consider explaining why you did not include Embase in your preliminary search.

 Please correct (PRIMSA) for (PRISMA).

 Consider including “perspectives” about the practice at your *phenomena of interest*, as you included it as a review question.

**Discussion:**

 The authors bring the term “receivers”, but in other places,  “recipients” is used. Personally, I consider “receivers” more appropriate, but I suggest you choose one term and be consistent about it.

Is the study design appropriate for the research question?

Yes

Is the rationale for, and objectives of, the study clearly described?

Yes

Are sufficient details of the methods provided to allow replication by others?

Yes

Are the datasets clearly presented in a useable and accessible format?

Not applicable

Reviewer Expertise:

Breastfeeding; breastfeeding medicine; human milk; donor human milk; neonatal nutrition

I confirm that I have read this submission and believe that I have an appropriate level of expertise to confirm that it is of an acceptable scientific standard.

## References

[1] : Development of LactaPedia: A lactation glossary for science and medicine. *Matern Child Nutr* .2020;16(3) : 10.1111/mcn.12969 e12969 10.1111/mcn.12969 32032481PMC7296804

# Reviewer report: Reviewer response for version 1

**DOI:** 10.21956/hrbopenres.15005.r34838

**Published:** 2023-07-19

**Authors:** Kaitlin Drouin

**Affiliations:** 1 Brigham & Women’s Hospital, Boston, USA

Thank you for the opportunity to read this timely and interesting paper, which describes the protocol for a planned mixed-methods systematic review to identify and summarize the populations, interests, and context related to the informal sharing of human milk in existing literature. This research will represent an important contribution to understanding this evolving yet long-standing phenomena.

 Below are some minor suggestions and comments to help the authors:

 Overall:

 JBI’s guidance and methods appear to be central to the work described in this paper. While this may be somewhat implicit, please consider adding a short description addressing why.

 Throughout, the authors use variations of the phrases “It has been affirmed”, “It is characterized,” “It has been stated,” “it has been documented”, “it has been articulated” when summarizing a cited source. Since it is implicit that a source stated/documented the content for which they are being cited, consider removing these statements for brevity.

 Introduction:

 Paragraph 1

- Please add citation(s) to support the sentence “Correspondingly, scientific research has confirmed that human milk is the perfect, unparalleled source of nutrition for infants.”
- In the second-to-last sentence, consider removing “which is suboptimal given the multiplicity of benefits derived from breastfeeding.” This is already stated in other ways elsewhere in the paragraph.

Paragraph 2

- The following points are re-stated here multiple times: 1) breastfeeding can have several meanings, 2) there has been a lack of consistency in the use of the term, and 3) using the term to define all types of human milk provision to an infant is inadequate. Consider consolidating the content on these points.

Methods:

 Search Strategy

- The author states three databases (CINAHL, Scopus, and MEDLINE) are used for the preliminary search using key terms. Then, after a more comprehensive search strategy is developed, four databases (CINAHL, Scopus, MEDLINE and Embase) are used. Consider describing why Embase was used in the second search and not the first.

Page 5, “Study selection”

- PRISMA is misspelled as “PRIMSA” in the second to last sentence

Page 5, “Assessment of methodological quality”

- Consider the following rewording to the second sentence for clarity: "Relevant components of the quantitative, qualitative and mixed-methods studies selected for retrieval will be assessed by two independent reviewers for methodological quality using standardized critical appraisal instruments from JBI SUMARI, dependent on study design (Aromataris & Munn, 2020).”

Data Extraction

- The authors state twice in this paragraph that the form represents the connection between the research studies and the SR. Consider removing one.

Data synthesis and integration

- Consider removing "and integrated together" from the last sentence, since integration is mentioned twice here.

Discussion:

- In the first sentence, it is unclear what "momentum" means. Authors may consider replacing “gaining momentum in recent times” with “rapidly growing and evolving” if that is what is meant here.

Is the study design appropriate for the research question?

Yes

Is the rationale for, and objectives of, the study clearly described?

Yes

Are sufficient details of the methods provided to allow replication by others?

Yes

Are the datasets clearly presented in a useable and accessible format?

Not applicable

Reviewer Expertise:

Infant feeding practices, infant nutrition and growth, human milk, donor human milk.

I confirm that I have read this submission and believe that I have an appropriate level of expertise to confirm that it is of an acceptable scientific standard.

# Reviewer report: Reviewer response for version 1

**DOI:** 10.21956/hrbopenres.15005.r33659

**Published:** 2023-05-26

**Authors:** Elizabeth J. O'Sullivan

**Affiliations:** 1 School of Biological and Health Sciences, Technological University Dublin, Dublin, Leinster, Ireland

Thank you for the opportunity to review this very interesting paper. This paper clearly describes a proposed protocol for a mixed-methods systematic review aiming to understand the factors associated with informal milk sharing and the perspectives of those involved in informal milk sharing. This is an understudied topic internationally, and also in the Irish context. The results of the proposed research will have public health and clinical relevance.

 I have some minor comments on the paper for the authors that they may find helpful:

- Throughout the entire paper, consider using an alternative word to “donating” and “donor” when describing informal milk sharing as I believe that most would consider “donating” to be associated with the milk bank. In terms of your review, using both words (and other variations) to search makes sense but in your description of the behaviour, I think distinguishing informal sharing from milk banking is important.
  - Consider “providing” or “provider” perhaps. So, this would mean you have providers and recipients, which distinguishes the practices from formal milk donation; see here for an example: DOI: 10.1177/0890334416651067 ^1^
  - Also, see here for a discussion around terminology ^2^ (could be referenced in the 2 ^nd^ paragraph of the introduction when the difficulty defining breastfeeding is discussed).
- Introduction:
  - Change complimentary foods to complementary foods.
  - Change “The SDGs comprise of 17…” to “The SDGs comprise 17…”
  - The sentence beginning “At this juncture” at the end of the second paragraph of the introduction is a little bit hard to follow, it feels unfinished. Also, I initially read “this juncture” to mean the present day rather than 1990.
  - In the third paragraph of the introduction, mother’s own milk is spelled out in full and abbreviated twice. Same for donor human milk.
  - The abbreviation HM is used mid-way through the third paragraph and is not used anywhere else in the paper. Consider writing human milk here.
  - The final sentence of the 3 ^rd^ paragraph begins with “In recent years…” and I think that this could be modified to indicate that it is the sharing of expressed milk that is new. The next section that goes on to describe wet nursing then builds on that.
  - It would be worth noting that there was an element of choice involved in wet nursing also… the British nobility would hire a wet nurse if the chose not to breastfeed themselves. So, it wasn’t solely about a necessity for child survival as might be the case of maternal death.
  - In the second paragraph on page 4, it would be worth referencing Sheela Geraghty and Sarah Keim, who have published work about milk sharing via the internet.
  - Third paragraph on page 4, IHMS is spelled out in full and abbreviated again even though it is on a previous page.
- Aim and review questions:
  - Should “informal human milk sharing” be abbreviated as IHMS throughout as was introduced in the introduction?
  - Here, and throughout, consider being more specific when you say “perspectives” of those involved in human milk sharing. Could you specify what exactly you are interested in exploring?
- Methods
  - In the description of the Population it is stated “Studies which include donors and/or receivers of human milk via milk banks will not be included.” I suggest a slight modification to “… via milk banks alone will not be included.” That way, you can include studies that may have explored both informal sharing and donating to milk banks in the same study. Same for those discussing purchasing of milk and wet nursing.
  - Could you expand on the rationale for not including wet nursing? It may be hard to distinguish in some cases. As in, there will be studies that comment on both types of behaviours.
  - In the “Phenomena” section, should it state “…recipients **of** human milk…”?
  - Also, in the “Phenomena” section, consider removal of the word “an” before “informal means.”
  - First paragraph on page 5, remove the word “of” after the word “comprise.”
  - Page 5, paragraph 2, consider removing the s of the word confirms.
  - In “Search strategy” the tense changes to past tense when describing the search that was undertaken, which is a bit distracting as the first two sentences are in the future tense.
  - Under “Assessment of methodological quality” is there a word missing in the following sentence: “Quantitative and qualitative and the relevant components of mixed-methods studies which are selected for retrieval will be assessed by two independent reviewers…”
  - Please review the following sentence for clarity: “Data extraction and synthesis will be performed on all studies, regardless of the results of their methodological quality results, where feasible.”

Is the study design appropriate for the research question?

Yes

Is the rationale for, and objectives of, the study clearly described?

Yes

Are sufficient details of the methods provided to allow replication by others?

Yes

Are the datasets clearly presented in a useable and accessible format?

Not applicable

Reviewer Expertise:

Nutrition, breastfeeding, infant feeding, breast milk expression, breast milk sharing.

I confirm that I have read this submission and believe that I have an appropriate level of expertise to confirm that it is of an acceptable scientific standard, however I have significant reservations, as outlined above.

## References

[1] : Informal Human Milk Sharing: A Qualitative Exploration of the Attitudes and Experiences of Mothers. *J Hum Lact* .2016;32(3) : 10.1177/0890334416651067 416-24 10.1177/0890334416651067 27271276

[2] : The Meaning of "Breastfeeding" Is Changing and So Must Our Language About It. *Breastfeed Med* .2017;12(9) : 10.1089/bfm.2017.0073 510-514 10.1089/bfm.2017.0073 28686471PMC5802247
